# Efficacy and safety of first-line chemotherapy combined with immune checkpoint inhibitors for extensive-stage small cell lung cancer patients: a real-world propensity score matching study

**DOI:** 10.3389/fimmu.2025.1562458

**Published:** 2025-08-13

**Authors:** Bin Jia, Chenyi Zhou, Fei Zhao, Xiaoru Song, Yuan Ding, Xiyin Wang, Boying Wu, Huina Wang, Quanman Hu, Shuaiyin Chen

**Affiliations:** ^1^ Department of Oncology, The First Affiliated Hospital of Zhengzhou University, Zhengzhou, China; ^2^ College of Public Health, Zhengzhou University, Zhengzhou, China

**Keywords:** ES-SCLC, chemotherapy, ICIs, PSM, COX, prognostic

## Abstract

**Background:**

extensive-stage small cell lung cancer (ES-SCLC) were the majority of SCLC patients. Recently the combination of chemotherapy with immune checkpoint inhibitors (ICIs) have emerged as the new first-line treatment standard for ES-SCLC. However, the specific patient populations that are most likely to benefit from this treatment remains to be clearly identified making the establishment of baseline biomarkers critical.

**Methods:**

We recruited ES-SCLC patients who were treated at the First Affiliated Hospital of Zhengzhou University and conducted a propensity score-matched analysis (PSM). And used the Kaplan-Meier (K-M) method and Cox proportional hazards regression to compare the survival outcomes. In addition, univariate and multivariate COX regression analyses were conducted to identify predictors.

**Results:**

After-PSM, chemotherapy group had a longer median overall survival (mOS) of 15.23 months (95%CI: 14.00-17.87) and hazard ratio (HR) of 0.576 (95% confidence interval (CI): 0.404-0.821), *P*=0.002), and the median progression free survival (mPFS) in the chemotherapy group was shorter: 6.05months (95%CI: 4.33-7.87), HR=0.707 (95%CI: 0.526 -0.950, *P*=0.021) compared to before PSM. Multivariate analysis confirmed that Eastern Cooperative Oncology Group performance status (ECOG PS) =1 (HR: 2.36, 95% CI: 1.38-4.03, *P*=0.002) and brain metastases (HR: 2.08, 95% CI: 1.05-4.14, *P*=0.038) were independent prognostic factors for PFS, and only systemic inflammation response index (SIRI)> 2.63 (HR: 0.06, 95% CI: 0.01-0.29, *P*<0.001) was an independent prognostic factor for OS.

**Conclusion:**

our findings indicate that incorporating ICIs into first-line chemotherapy significantly improves PFS and OS in ES-SCLC patients, while maintaining safety. Moreover, poor ECOG PS, brain metastases, and high SIRI at baseline may serve as valuable prognostic indicators for disease progression and survival in ES-SCLC patients undergoing first-line chemotherapy plus ICIs. It is worth noting that these findings should be interpreted as hypothesis-generating, not definitive clinical conclusions.

## 1 Introduction

Lung cancer is the most prevalent and deadliest form of cancer both globally and in China (1). In approximately 15% of lung cancer cases, the diagnosis is small cell lung cancer (SCLC). This subtype of lung cancer is defined by a high proliferation rate, early development of metastasis, and an unfavorable prognosis (2). Consequently, the 5-year survival rate for patients diagnosed with SCLC is reported to be only 5% (3). Approximately 30% of SCLC cases are classified as limited-stage SCLC (LS-SCLC), while the majority are extensive-stage SCLC (ES-SCLC) (4). Notably, many SCLC patients are diagnosed with ES-SCLC at the initial presentation, in contrast to those with LS-SCLC, studies have demonstrated that ES-SCLC has a shorter median overall survival (mOS) and a less favorable prognosis, with a 2-year survival rate of merely 2% (5, 6).

Major breakthrough in the treatment of SCLC have been limited over an extended period. For ES-SCLC, the standard therapeutic approach remains systemic chemotherapy. This regimen typically comprises platinum-based compounds in conjunction with etoposide (7). ES-SCLC demonstrates a high degree of initial sensitivity to chemotherapy, resulting in notable response rates. However, this condition is also marked by a high recurrence rate and an unfavorable prognosis. The mOS with standard therapy is only 9–10 months, underscoring the need for novel therapeutic approaches (8). A recent Phase 3 clinical trial demonstrated that first-line durvalumab (a programmed death-ligand 1 (PDL-1) inhibitor) in combination with platinum-etoposid significantly improved OS was observed in patients with ES-SCLC treated with the combination regimen compared to platinum-etoposide alone, and the mOS was 12.9 months versus 10.5 months, respectively. These findings suggest that the combination of durvalumab and etoposide may represent a new standard of care for first-line treatment of ES-SCLC (9). Similarly, another Phase 3 clinical trial showed statistically significant clinical benefits and a manageable safety profile when combining a tirellizumab (programmed death 1 (PD-1) inhibitor) with chemotherapy. Therefore, the addition of immune checkpoint inhibitors (ICIs) (including PD-1 and PDL-1) to platinum-based chemotherapy has altered the treatment paradigm for ES-SCLC. Over five years of follow-up, chemotherapy combined ICIs with have shown sustained benefits, with a significant increase in five-year survival rates by 12% (10).

Currently, the Chinese Consensus on Immunotherapy for Small Cell Lung Cancer (2024 edition) has endorsed the combination of chemotherapy with ICIs as the new first-line treatment standard for ES-SCLC (11). However, the majority of patients experience disease progression within 6 months of initiating first-line therapy. To date, there is no definitive and reliable method to identify patients who are most likely to benefit from this treatment approach, making it crucial to establish baseline biomarkers. Recently, several novel immunoinflammatory and nutritional indicators have been utilized in combination therapy to predict prognosis in non-small cell lung cancer (NSCLC), such as the prognostic nutritional index (PNI), platelet-to-lymphocyte ratio (PLR), lung immune prognostic index (LIPI), neutrophil-to-lymphocyte ratio (NLR) and lymphocyte-to-monocyte ratio (LMR) (12–14). However, limited studies have explored the use of these indicators as biomarkers for predicting the prognosis of ES-SCLC patients treated with ICIs combined with chemotherapy.

In this study, we evaluated the efficacy of ICIs in combination with chemotherapy compared to chemotherapy alone in the clinical management of ES-SCLC, focusing on endpoints such as OS, progression free survival (PFS), disease control rate (DCR), objective response rate (ORR), and treatment-related adverse events (AEs), to provide a reference for clinicians. To ensure the comparability of baseline characteristics, propensity score matching (PSM) was employed. Additionally, to identify independent prognostic factors among patients treated with chemotherapy plus ICIs for ES-SCLC, univariate and multivariate COX regression analyses were performed. These factors may encompass demographic characteristics, clinical features at initial diagnosis, tumor markers, serum immunity, inflammation, and nutritional indicators.

## 2 Methods

### 2.1 ES-SCLC patients selection

This retrospective study was conducted in accordance with the revised Declaration of Helsinki and received approval from the Ethics Committee of the First Affiliated Hospital of Zhengzhou University (Ethics Approval Number: 2024-KY-0189-002, Ethics Approval Date: 2024-03-11). Patients diagnosed with SCLC who received treatment at the First Affiliated Hospital of Zhengzhou University between January 2020 and April 2022 were included in this study. Diagnosis of SCLC was confirmed through definitive cytological and histological evidence, verified by the clinician responsible for data collection. Standard first-line chemotherapy comprised etoposide plus platinum, with or without ICIs. ES-SCLC was defined according to the criteria established by the Veterans Administration Lung Study Group (VALG) (15). Patients with ES-SCLC who received only 1–2 cycles of treatment, had a history of other malignancies, underwent surgical intervention, or patients with an Eastern Cooperative Oncology Group performance status (ECOG PS) greater than 1 were excluded from the study. The follow-up period extended until September 1, 2024. Due to the retrospective nature of this study, informed consent was waived. All patient personal information was maintained with strict confidentiality.

### 2.2 Data collection and evaluation

To evaluate the efficacy of first-line chemotherapy with or without ICIs in patients with ES-SCLC, we utilized the Kaplan-Meier (K-M) method to PFS and OS. Furthermore, imaging assessments were performed for each treatment regimen, and responses were evaluated according to the Response Evaluation Criteria In Solid Tumors (RECIST) version 1.1. This allowed us to assess the therapeutic outcomes during first-line treatment and subsequently calculate the ORR and DCR. Specifically, ORR was calculated as (complete response (CR) + partial response (PR))/(CR + PR + stable disease (SD) + progressive disease (PD)), while DCR was determined as (CR + PR + SD)/(CR + PR + SD + PD). Adverse events (immune-related (ir) AEs and other adverse drug reactions) graded using the National Cancer Institute’s Common Terminology Criteria for Adverse Events version 5.0, and irAEs mainly included suggestive of immune-mediated events (e.g., skin rash, pneumonitis, thyroid dysfunction, nephritis, hepatitis, and hypophysitis).

In this study, we collected comprehensive baseline information prior to first-line treatment, including demographic data (age, sex), lifestyle factors (smoking history), performance status (ECOG PS), and radiotherapy history. Additionally, we recorded the baseline TNM stage and laboratory test results, such as leukocytes, lymphocytes, monocytes, neutrophils, lactate dehydrogenase (LDH), platelets, serum albumin, and various tumor markers. Based on these data, we calculated several composite indicators:

The NLR is calculated as the neutrophil count divided by the lymphocyte count. The derived neutrophil-to-lymphocyte ratio (dNLR) is determined by dividing the neutrophil count by the difference between the white blood cell count and the neutrophil count. The LMR is obtained by dividing the lymphocyte count by the monocyte count. The PLR is computed as the platelet count divided by the lymphocyte count. The systemic immune-inflammation index (SII) is defined as the product of the platelet count and the neutrophil count, divided by the lymphocyte count. The systemic inflammation response index (SIRI) is calculated as the product of the neutrophil count and the monocyte count, divided by the lymphocyte count. The PNI is given by the sum of serum albumin concentration (in g/L) and five times the total number of peripheral blood lymphocytes. The LIPI is assessed using baseline LDH levels and dNLR to categorize patients into three prognostic groups: poor (dNLR > 3 and LDH > ULN), intermediate (dNLR > 3 or LDH > ULN) and good (dNLR ≤ 3 and LDH ≤ upper limit of normal [ULN]). In addition, ULN was used to convert LDH and tumor markers into categorical variables, and the best cutoff values for SII, LMR, PLR, SIRI, PNI, and PAR were determined by the “surv_cutpoint” function.

### 2.3 Statistical analysis

Descriptive analysis (frequency and percentage) was conducted to summarize the baseline characteristics of ES-SCLC patients across different treatment groups. Statistical comparisons were performed using the χ2 test and the Kruskal-Wallis test as appropriate. To analyze survival data, the K-M method was employed, and differences in survival curves were assessed using the log-rank test.

Subgroup analyses were based on several variables. To mitigate the risk of false positive findings due to multiple comparisons across these subgroups, we applied false discovery rate (FDR) correction using the Benjamini-Hochberg procedure to all subgroup interaction P-values and within-subgroup treatment effect P-values.

PSM was performed using a 2:1 optimal matching method with a tolerance level of 0.05. The covariates included in the matching process were age, sex, smoking status, ECOG PS, Tumor primary site, radiotherapy, tumor size, node metastasis, extrathoracic metastasis, adrenal gland metastasis, brain metastasis, bone metastasis, liver metastasis, pleural metastasis, other sites, total bilirubin and uric acid. A logistic regression model was used to estimate the propensity scores and the standardized differences (SMD) and love plot are used to assess the balance between the two groups.

To identify predictors of OS and PFS in ES-SCLC patients treated with chemotherapy combined with ICIs, univariate and multivariate COX regression analyses were conducted to evaluate the data. Variables that exhibited a univariate *P*-value < 0.05 were included in the multivariate COX regression analysis using bidirectional stepwise selection. All statistical analyses were performed using R software (version 4.4.2) and SPSS version 25.0, with a significance level set at *P* < 0.05.

## 3 Results

### 3.1 Basic characteristics of ES-SCLC patients

**Figure 1:**
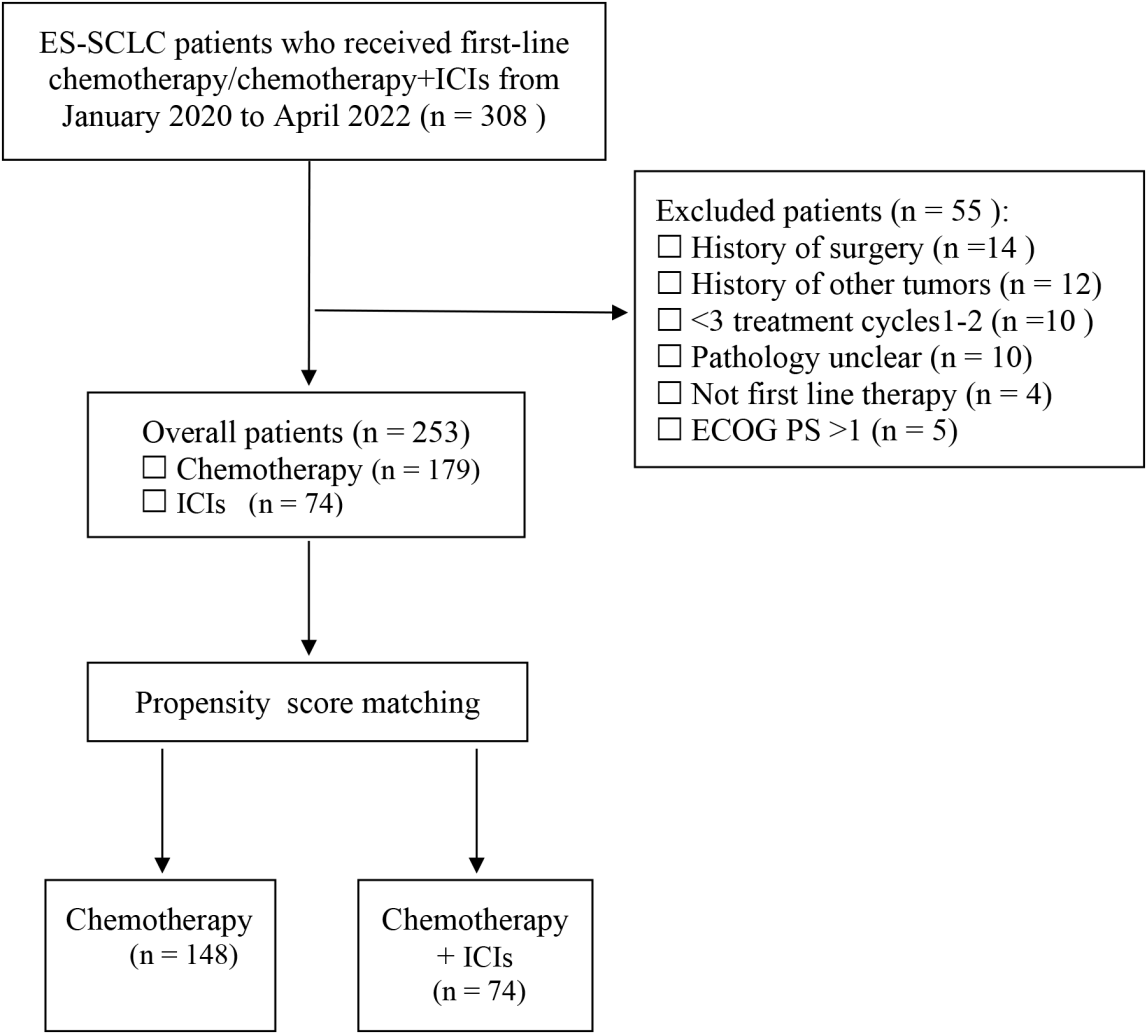
Diagram of the patient’s selection process. SCLC, Small cell lung cancer; ES, extensive-stage; ICIs, immune checkpoint inhibitors.

Initially, a total of 308 ES-SCLC patients were identified as shown in **Figure 1**. After applying the inclusion and exclusion criteria, 253 ES-SCLC patients were included for evaluation. Among these, 179 patients received chemotherapy alone while 74 patients received chemotherapy combined with ICIs. **Table 1** summarizes the baseline characteristics of the 253 patients: 119 (47.04%) were aged ≥65 years, 77.47% were male, and 60.87% had a history of smoking. The majority of tumor size (69.87%) were larger than 5 cm, and most metastatic sites were extrathoracic (183 patients). The top three metastatic sites were bone (71 patients), liver (68 patients), and brain (34 patients). Prior to PSM, some baseline characteristics differed between the chemotherapy and chemotherapy combined with ICIs. For instance, the ECOG SMD was 0.1266, which was greater than 0.1, indicating an imbalance between the two groups. After PSM, the ECOG SMD decreased to 0.0878. Moreover, the SMDs of other covariates also showed a certain degree of decrease (**Supplementary Table S1**, **Supplementary Figures S1**, **S2**).

**Table 1:** Baseline characteristics before and after propensity score matching according to first-line therapy (chemotherapy/chemotherapy+ICIs).

Variables	Subcategories	n (%) All patients (n = 253)	Before matching n (%)		After matching n (%)			
			Chemotherapy (n = 179)	Chemotherapy +ICIs (n = 74)	*P*	Chemotherapy (n =148)	Chemotherapy +ICIs (n = 74)	*P*
Age	<65	134 (52.96)	92 (51.40)	42 (56.76)	0.437	76 (51.35)	42 (56.76)	0.447
	≥65	119 (47.04)	87 (48.60)	32 (42.24)		72 (48.65)	32 (42.24)	
Sex	Male	196 (77.47)	142 (79.33)	54 (72.97)	0.271	115 (77.70)	54 (72.97)	0.436
	Female	57 (22.53)	37 (20.67)	20 (27.03)		33 (22.30)	20 (27.03)	
Smoke	NO	99 (39.13)	69 (38.55)	30 (40.54)	0.768	54 (36.49)	30 (40.54)	0.557
	YES	154 (60.87)	110 (61.45)	44 (59.46)		94 (63.51)	44 (59.46)	
ECOG PS	0	97 (38.34)	62 (34.64)	35 (47.30)	0.060	57 (38.51)	35 (47.30)	0.210
	1	156 (61.66)	117 (65.36)	39 (52.70)		91 (61.49)	39 (52.70)	
Primary site	Left	113 (44.67)	79 (44.13)	34 (45.95)	0.792	63 (42.57)	34 (45.95)	0.632
	Right	135 (53.36)	97 (54.19)	40 (54.05)		85 (57.43)	40 (54.05)	
Radiotherapy	NO	170 (67.19)	120 (67.04)	50 (67.37)	0.935	102 (68.92)	50 (67.37)	0.838
	YES	83 (34.78)	59 (32.96)	24 (32.43)		46 (31.08)	24 (32.43)	
Tumor size	< 5cm	88 (65.22)	58 (32.40)	30 (40.54)	0.216	55 (37.16)	30 (40.54)	0.625
	≥5cm	165 (69.87)	121 (67.60)	44 (59.46)		93 (62.84)	44 (59.46)	
Node metastases	NO/ LOCAL	149 (58.89)	107 (59.78)	42 (56.76)	0.657	90 (60.81)	42 (56.76)	0.562
	Distant	104 (41.11)	72 (40.22)	32 (43.24)		58 (39.19)	32 (43.24)	
Extrathoracic metastases	NO	70 (27.67)	53 (29.61)	17 (22.97)	0.283	39 (26.35)	17 (22.97)	0.585
	YES	183 (72.33)	126 (70.39)	57 (77.03)		109 (73.65)	57 (77.03)	
Adrenalgland metastases	NO	223 (88.14)	157 (87.71)	66 (89.19)	0.741	129 (87.16)	66 (89.19)	0.663
	YES	20 (11.86)	22 (12.29)	8 (10.81)		19 (12.84)	8 (10.81)	
Brain metastases	NO	219 (86.56)	155 (86.59)	64 (86.49)	0.982	129 (87.16)	64 (86.49)	0.888
	YES	34 (13.44)	24 (13.41)	10 (13.51)		19 (12.84)	10 (13.51)	
Bone metastases	NO	182 (71.94)	127 (70.95)	55 (74.32)	0.587	106 (71.62)	55 (74.32)	0.671
	YES	71 (28.06)	52 (29.05)	19 (25.68)		42 (28.38)	19 (25.68)	
Liver metastases	NO	185 (73.12)	132 (73.74)	53 (71.62)	0.729	106 (71.62)	53 (71.62)	1.000
	YES	68 (26.88)	47 (26.26)	21 (28.38)		42 (28.38)	21 (28.38)	
Pleural metastases	NO	237 (93.68)	169 (94.41)	68 (91.89)	0.453	139 (93.92)	68 (91.89)	0.571
	YES	16 (6.32)	10 (5.59)	6 (8.11)		9 (6.08)	6 (8.11)	
Other sites	NO	233 (91.30)	168 (93.85)	63 (85.14)	0.025	137 (92.57)	63 (85.14)	0.081
	YES	22 (8.70)	11 (6.15)	11 (14.86)		11 (7.43)	11 (14.86)	
Total bilirubin	Normal	220 (87.96)	153 (85.47)	67 (90.54)	0.276	131 (88.51)	67 (90.54)	0.647
	Unnormal	33 (13.04)	26 (14.53)	7 (9.46)		17 (11.49)	7 (9.46)	
Uric acid	Normal	186 (73.52)	129 (72.07)	57 (77.03)	0.416	108 (72.97)	57 (77.03)	0.515
	Unnormal	67 (26.48)	50 (27.93)	17 (22.97)		40 (27.03)	17 (22.97)	

SCLC, Small cell lung cancer; ICIs, immune checkpoint inhibitors; ECOG PS, Eastern Cooperative Oncology Group performance status.

### 3.2 Survival outcome before and after PSM

**Figure 2:**
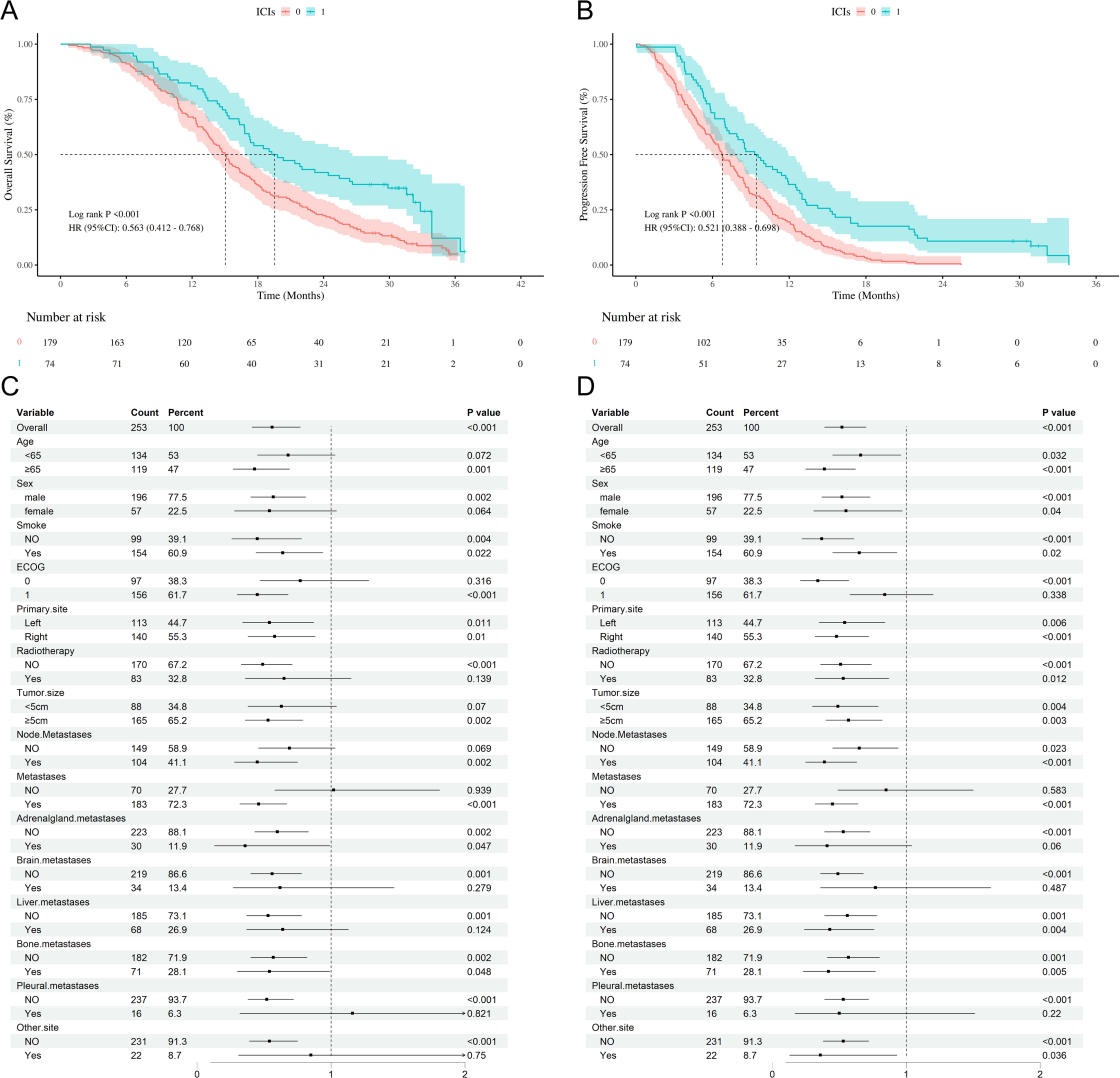
Survival outcomes of chemotherapy/chemotherapy +ICIs. **(A)** Kaplan-Meier curves of OS stratified by chemotherapy/chemotherapy +ICIs; **(B)** Kaplan-Meier curves of PFS stratified by chemotherapy/chemotherapy +ICIs; **(C)** Forest plot of subgroup analysis of OS; **(D)** Forest plot of subgroup analysis of PFS; OS, overall survival; PFS, progression free survival; SCLC, Small cell lung cancer; ICIs, immune checkpoint inhibitors; HR, hazard ratio; CI, confidence interval; ECOG PS, Eastern Cooperative Oncology Group performance status.

Before PSM, we used K-M curves to analyze the survival outcomes comparing the chemotherapy group and the chemotherapy plus ICIs group, as illustrated in **Figure 2A**. The mOS in the chemotherapy plus ICIs group was 19.54 months (95% CI: 16.80-26.63), compared to 15.03 months (95% CI: 13.80-16.37) in the chemotherapy group, with a hazard ratio (HR) of 0.563 (95% CI: 0.412-0.768, *P* < 0.001). Similarly, the median mPFS in the chemotherapy plus ICIs group was 9.44 months (95% CI: 7.30-11.93), compared to 6.03 months (95% CI: 4.70-7.87) in the chemotherapy group, with an HR of 0.521 (95% CI: 0.388-0.698, *P* < 0.001) (**Figure 2B**).

We then conducted a subgroup analysis based on baseline information, as shown in **Figures 2C, D**, **Supplementary Table S2**. Even though chemotherapy combined with ICIs was beneficial in both OS and PFS, some subgroups of people did not show statistically significant benefit, regardless of OS or PFS. For example, Metastases = NO (OS: FDR-adjusted *P*=0.939, PFS: FDR-adjusted *P*=0.583); Brain metastases (OS: FDR-adjusted *P*=0.332, PFS: FDR-adjusted *P*=0.516). Pleural metastases (OS: FDR-adjusted *P*=0.855, PFS: FDR-adjusted *P*=0.054). Finally, we took patients receiving chemotherapy combined with ICIs as the baseline, and based on the baseline information, 2:1 matching was performed for ES-SCLC patients receiving chemotherapy alone.

**Figure 3:**
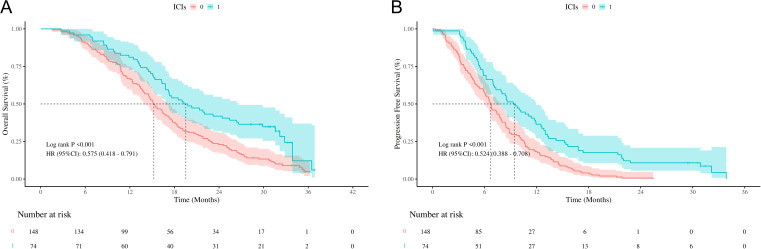
Survival outcomes of chemotherapy/chemotherapy +ICIs after PSM. **(A)** Kaplan-Meier curves of OS stratified by chemotherapy/chemotherapy +ICIs after PSM; **(B)** Kaplan-Meier curves of PFS stratified by chemotherapy/chemotherapy +ICIs after PSM; OS, overall survival; PFS, progression free survival; PSM, propensity score matching; ICIs, immune checkpoint inhibitors; HR, hazard ratio; CI, confidence interval.

K-M curves demonstrated results consistent with those before PSM, the mOS in the chemotherapy group was 15.23 months (95% CI: 13.90-17.23), compared to 19.54 months (95% CI: 16.80-26.63) in the chemotherapy plus ICIs group, with a hazard ratio (HR) of 0.575 (95% CI: 0.418-0.791, *P* < 0.001, **Figure 3A**). Similarly, the median mPFS in the chemotherapy was 6.71 months (95% CI: 5.97-7.73), compared to 9.44 months (95% CI: 7.30-11.93) in the chemotherapy plus ICIs group, with an HR of 0.524 (95% CI: 0.388-0.708, P < 0.001, **Figure 3B**).

### 3.3 Response and treatment-related AEs

**Figure 4:**
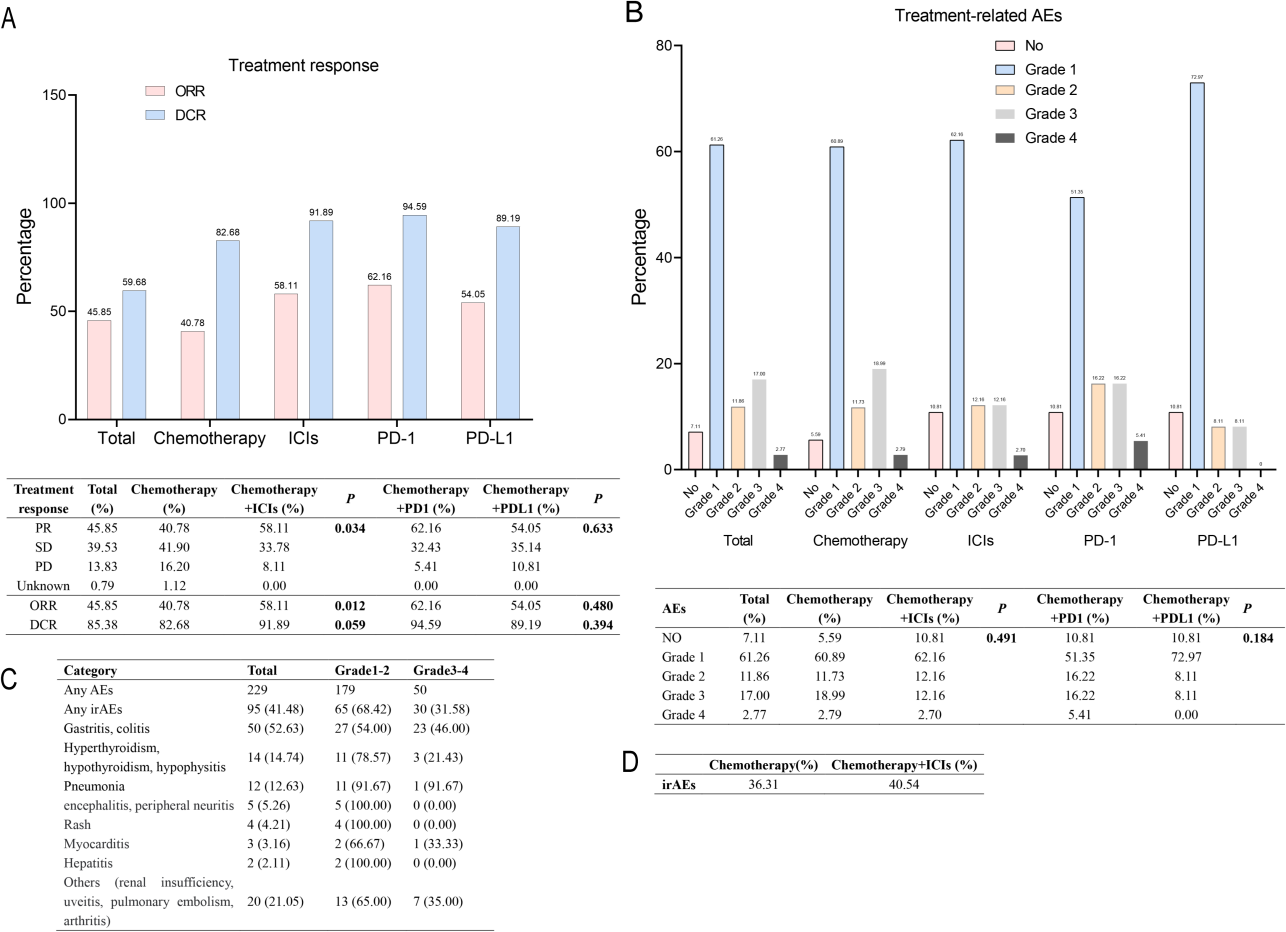
Tumor response and treatment-related AEs in all ES-SCLC patients. **(A)** Tumor response in all ES-SCLC patients. **(B)** Treatment-related AEs in all ES-SCLC patients. **(C)** irAEs in all ES-SCLC patients **(D)** irAEs in chemotherapy group and chemotherapy plus ICIs group ES, extensive-stage; AEs, adverse events; ICIs, immune checkpoint inhibitors; PR, partial response; SD, stable disease; ORR, objective response rate; DCR, disease control rate; PD-1, programmed cell death protein 1; PDL -1, programmed cell death ligand 1. irAEs, immune-related adverse events.

Treatment response was evaluated in 253 patients, as showed in **Figure 4A**. The DCR and ORR for all ES-SCLC patients were 85.38% and 45.85%, respectively. Specifically, the DCR and ORR for the chemotherapy group were 82.68% and 40.78%, while those for the chemotherapy plus ICIs group were 91.89% and 58.11%, respectively. The difference in ORR between the two groups was statistically significant (*P*=0.012), whereas the difference in DCR was not (*P=*0.059). Additionally, there were statistically significant differences between the two groups in PR, SD, and PD rates (*P*=0.034). Subsequently, we compared the ORR and DCR between PD-1 and PDL -1 inhibitors, finding no statistically significant differences (ORR: *P*=0.480; DCR: *P*=0.394; overall: *P*=0.633).

Regarding treatment-related AEs, 92.89% of the 253 patients experienced AEs, with the majority being Grade 1 (61.26%) and a smallest proportion being Grade 4 (2.77%). No Grade 5 events, which are considered life-threatening, were reported. In contrast to the treatment response, there was no significant difference in AEs incidence between the chemotherapy group and the chemotherapy plus ICIs group (*P*=0.491), nor between PD-1 and PDL-1 (*P*=0.184, **Figure 4B**). Then we performed a reanalysis of the irAEs in all SCLC patients. The results are presented in **Figures 4C, D**. Among all SCLC patients who experienced AEs, 41.48% had irAEs. Gastritis and colitis were the most prevalent, accounting for 52.53%, and the irAEs in the chemotherapy plus ICIs group was 40.54%, which was higher than that in the chemotherapy alone group (36.31%).

### 3.4 Prognostic factors of OS and PFS in ES-SCLC patients treated with chemotherapy plus ICIs

Univariate and multivariate COX regression analyses were conducted for the chemotherapy plus ICIs treatment group, with clinical outcomes defined as death and disease progression. The aim was to identify baseline characteristics, serum inflammatory markers, and tumor markers associated with survival. The results are summarized in **Table 2**. The univariate analysis revealed that ECOG PS=1 (HR: 2.37, 95% CI: 1.38-4.06, *P*=0.002) and brain metastases (HR: 2.10, 95% CI: 1.05-4.18, *P*=0.036) were associated with PFS in patients receiving first-line chemotherapy plus ICIs. Multivariate analysis confirmed that ECOG PS=1 (HR: 2.36, 95% CI: 1.38-4.03, *P*=0.002) and brain metastases (HR: 2.08, 95% CI: 1.05-4.14, *P*=0.038) were independent prognostic factors for PFS.

**Table 2:** Univariate and multivariate analyses of progression-free survival and overall survival in ES-SCLC patients who received first-line chemotherapy +ICIs.

Variables	Subcategories		PFS-Univariate analysis		PFS-Multivariae analysis		OS- Univariate analysis		OS- Multivariate analysis
		*P*	HR (95%CI)	*P*	HR (95%CI)	*P*	HR (95%CI)	*P*	HR (95%CI)
Age	<65								
	≥65	0.276	0.76 (0.47 ~ 1.24)			0.280	0.74 (0.43 ~ 1.28)		
Sex	Male								
	Female	0.920	0.97 (0.57 ~ 1.67)			0.956	0.98 (0.52 ~ 1.85)		
Smoke	NO								
	YES	0.179	1.40 (0.86 ~ 2.28)			0.106	1.60 (0.91 ~ 2.82)		
ECOG PS	0								
	1	0.002	2.37 (1.38 ~ 4.06)	0.002	2.36 (1.38 ~ 4.03)	0.428	1.25 (0.72 ~ 2.17)		
Primary site	Left								
	Right	0.639	1.12 (0.69 ~ 1.82)			0.983	1.01 (0.59 ~ 1.72)		
Radiotherapy	NO								
	YES	0.706	1.10 (0.66 ~ 1.83)			0.302	0.73 (0.40 ~ 1.33)		
Tumor size	< 5cm								
	≥5cm	0.452	1.21 (0.74 ~ 1.98)			0.438	0.81 (0.47 ~ 1.39)		
Node metastases	NO/LOCAL								
	Distant	0.113	0.67 (0.41 ~ 1.10)			0.064	0.59 (0.34 ~ 1.03)		
Extrathoracic metastases	NO								
	YES	0.201	0.69 (0.40 ~ 1.21)			0.108	0.62 (0.34 ~ 1.11)		
Adrenalgland metastases	NO								
	YES	0.880	1.06 (0.48 ~ 2.33)			0.962	1.02 (0.41 ~ 2.58)		
Brain metastases	NO								
	YES	0.036	2.10 (1.05 ~ 4.18)	0.037	2.08 (1.05 ~ 4.14)	0.953	0.98 (0.44 ~ 2.18)		
Bone metastases	NO								
	YES	0.408	0.79 (0.45 ~ 1.39)			0.631	1.16 (0.63 ~ 2.15)		
Liver metastases	NO								
	YES	0.771	0.92 (0.54 ~ 1.57)			0.108	1.61 (0.90 ~ 2.88)		
Pleural metastases	NO								
	YES	0.751	0.87 (0.38 ~ 2.03)			0.862	1.09 (0.39 ~ 3.05)		
Other sites	NO								
	YES	0.897	0.95 (0.47 ~ 1.93)			0.754	1.14 (0.51 ~ 2.52)		
ICIs	PD-1								
	PD-L1	0.464	1.19 (0.74 ~ 1.92)			0.943	0.98 (0.57-1.68)		
LDH	≤245								
	>245	0.831	1.07 (0.56 ~ 2.07)			0.707	1.17 (0.52 ~ 2.60)		
CEA	≤5								
	>5	0.725	1.10 (0.65 ~ 1.85)			0.667	1.14 (0.63 ~ 2.04)		
Cyfran21	≤3.3								
	>3.3	0.193	1.52 (0.81 ~ 2.86)			0.915	0.96 (0.48 ~ 1.95)		
CA125	≤35								
	>35	0.805	0.93 (0.54 ~ 1.61)			0.469	1.26 (0.67 ~ 2.35)		
CA19-9	≤37								
	>37	0.174	1.58 (0.82 ~ 3.05)			0.627	0.82 (0.37 ~ 1.81)		
CA72-4	≤7								
	>7	0.652	0.82 (0.34 ~ 1.95)			0.029	2.71 (1.11 ~ 6.62)		
NSE	≤16.3								
	>16.3	0.647	1.19 (0.56 ~ 2.54)			0.388	1.51 (0.59 ~ 3.88)		
SII	≤146.11								
	>146.11	0.648	0.88 (0.50 ~ 1.54)			0.028	0.44 (0.22 ~ 0.92)		
NLR	≤4								
	>4	0.917	0.97 (0.57 ~ 1.67)			0.047	0.50 (0.25 ~ 0.99)	0.080	2.61 (0.89 ~7.66)
dNLR	≤3								
	>3	0.617	0.85 (0.44 ~ 1.62)			0.176	0.58 (0.26 ~ 1.28)		
LIPI	Good								
	Intermediate	0.837	0.93 (0.45 ~ 1.90)			0.887	1.06 (0.45 ~ 2.50)		
	Poor	0.303	1.77 (0.60 ~ 5.25)			0.940	1.05 (0.30 ~ 3.65)		
LMR	≤2.01								
	>2.01	0.974	0.99 (0.56 ~ 1.74)			0.008	2.90 (1.33 ~ 6.34)		
PLR	≤288.42								
	>288.42	0.965	1.02 (0.48 ~ 2.13)			0.057	0.36 (0.13 ~ 1.03)		
SIRI	≤2.63								
	>2.63	0.965	0.99 (0.56 ~ 1.75)			<0.001	0.22 (0.09 ~ 0.53)	<0.001	0.06 (0.01 ~ 0.29)
PAR	≤6.00								
	>6.00	0.668	1.15 (0.61 ~ 2.15)			0.055	0.46 (0.20 ~ 1.02)		
PNI	≤40.45								
	>40.45	0.120	0.53 (0.24 ~ 1.18)			0.151	2.00 (0.78 ~ 5.17)		

OS, overall survival; PFS, progression free survival; ICIs, immune checkpoint inhibitors. HR, hazard ratio; CI, confidence interval; ES, extensive-stage; PD-1, programmed cell death protein 1; PD-L1, programmed cell death ligand 1; ECOG PS, Eastern Cooperative Oncology Group performance status; LDH, lactate dehydrogenase; NLR, neutrophil-to-lymphocyte ratio; dNLR, derived Neutrophil to Lymphocyte Ratio; LIPI, lung immune prognostic index; LMR, lymphocyte to monocyte ratio; PLR, platelet to lymphocyte ratio; PAR, platelet to albumin ratio; PNI, prognostic nutrition index; SII, systemic immune-inflammation index; SIRI, systemic inflammation response index; CEA, carcinoembryonic antigen; NSE, Neuron-specific enolase; CA125, Cancer antigen 125; CA153, Carbohydrate antinegen 15-3; Cyfran21, cytokeratin 19 fragments.

In contrast, the univariate analysis for OS showed that elevated CA72-4 (>7 U/mL, HR: 2.71, 95% CI: 1.11-6.62, *P*=0.029), SII > 146.11 (HR: 0.44, 95% CI: 0.22-0.92, *P*=0.028), NLR > 4 (HR: 0.50, 95% CI: 0.25-0.99, *P*=0.047), LMR> 2.01 (HR: 2.90, 95% CI: 1.33-6.34, *P*=0.008), and SIRI > 2.63 (HR: 0.29, 95% CI: 0.09-0.31, *P*<0.001) were associated with OS. However, multivariate analysis indicated that only SIRI > 2.63 (HR: 0.06, 95% CI: 0.01-0.29, *P*<0.001) was an independent prognostic factor for OS.

**Figure 5:**
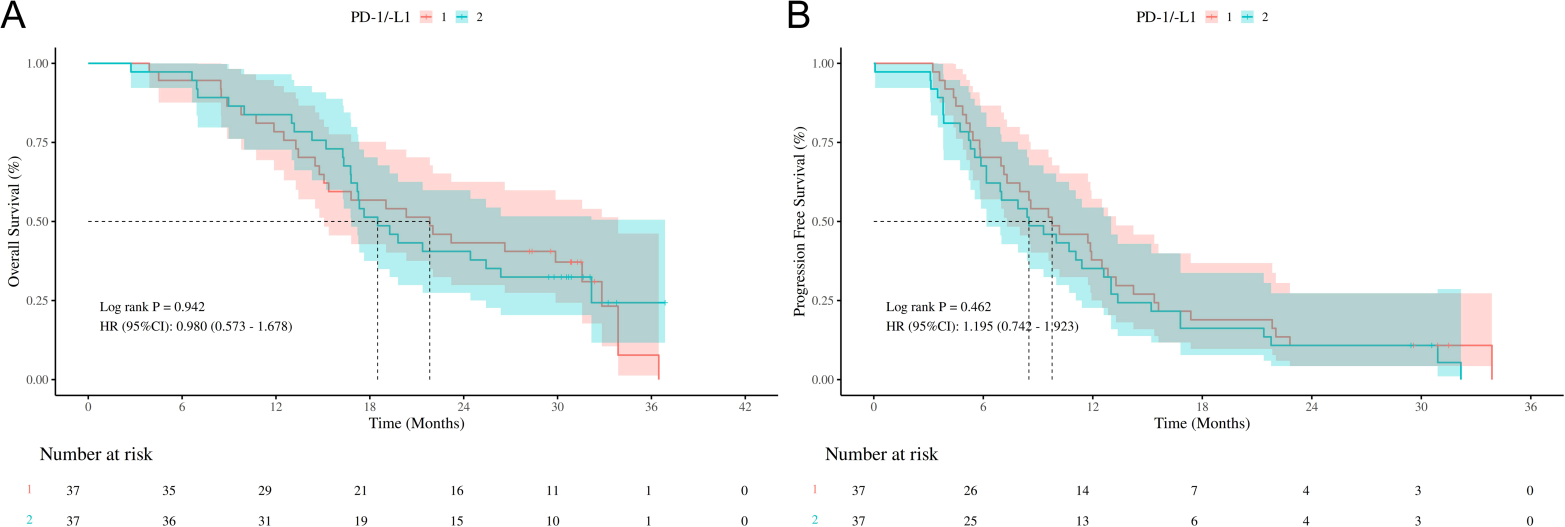
Survival outcomes of different ICIs (PD-1/PDL-1) **(A)** Kaplan-Meier curves of OS stratified by different ICIs (PD-1/PDL-1); **(B)** Kaplan-Meier curves of PFS stratified by different ICIs (PD-1/PDL-1); OS, overall survival; PFS, progression free survival; ICIs, immune checkpoint inhibitors. HR, hazard ratio; CI, confidence interval; PD-1, programmed cell death protein 1; PDL-1, programmed cell death ligand 1.

In addition, survival analysis was conducted to compare the outcomes of ES-SCLC patients treated with PD-1 inhibitors versus those treated with PDL-1 inhibitors. As shown in **Figure 5A**, the HR for OS between the PD-1 and PDL-1 groups was 0.980 (95% CI: 0.573-1.678, *P*=0.942), but the wide CI crossing the null value (HR=1) precludes definitive conclusions regarding equivalence or difference between groups.

Similarly, as depicted in **Figure 5B**, the HR for PFS between the two groups was 1.195 (95% CI: 0.742-1.923, *P*=0.462), suggesting inconclusive evidence due to the imprecise estimate reflected by the wide CI.

## 4 Discussion

ES-SCLC accounts for the vast majority of all SCLC patients and is characterized by poor prognosis and limited treatment. In the past 30 years, the main treatment has been chemotherapy based on platinum-based drugs (etoposide + cisplatin/carboplatin), but the long-term survival is low, 5-year survival rate <7% (16). However, the application of some ICIs (such as durvalumab and atezolizumab) in first-line treatment has prolonged the OS of ES-SCLC patients, which brings a new dawn to the long-term unchanged treatment mode of ES-SCLC and is also a milestone in the change of treatment mode. These include CASPIAN (9), IMpower133 (17), ASTRUM005 (18), Checkmate032 (19), and Keynote028 (20).

Based on the more encouraging and effective results of multi clinical trials, many countries have adopted ICIs (PD-1/PDL-1 inhibitor) combined with chemotherapy as the first-line treatment for ES-SCLC and included it in the relevant treatment guidelines (11, 21). However, in the clinical management of ES-SCLC, the efficacy of first-line chemotherapy in combination with ICIs remains an area worthy of further investigation. And the role of baseline data (mainly tumor markers, serum immunoinflammatory and nutritional indicators, etc.) in evaluating the prognosis of patients with ES-SCLC treated by such methods is still unclear. To investigate the efficacy and safety of first-line chemotherapy combined with ICIs in patients with ES-SCLC, as well as to identify biomarkers associated with patient prognosis, we conducted a retrospective matched-pair analysis.

In this retrospective study, we analyzed data from super-large medical centers in central China. The results indicated that the K-M curves before and after PSM demonstrated that patients with ES-SCLC who received ICIs combined with chemotherapy had significantly higher OS and PFS, along with fewer AEs. Notably, although some baseline covariates were no longer significantly different before-PSM, they continued to influence the final outcomes. After-PSM, patients with ES-SCLC who received only chemotherapy experienced a longer mOS and mPFS, likely due to diminished differences in baseline covariates and confounders among treatment groups (22). This aligns with clinical trial findings: durvalumab, a PD-1 inhibitor, combined with chemotherapy significantly increased mOS and mPFS, while AEs did not differ significantly from chemotherapy alone, occurring at rates of 32% and 36%, respectively (9).Meanwhile, our data indicate that the ORR in the chemotherapy plus ICIs group was 58.11%, which was significantly higher than that in the chemotherapy group (40.7%). This finding is consistent with the results of the CASPIAN trial: the chemotherapy plus durvalumab group demonstrated a significant improvement in the ORR compared to the chemotherapy group (23). Regarding DCR, the 91.89% in the chemotherapy plus CIs group was higher than the 82.68% in the chemotherapy group, which is consistent with the findings of the IMpower133 trial (24).

Our subgroup analysis revealed that patients without extrathoracic metastases, brain metastases, or pleural metastases did not benefit from the combination therapy, whereas most other subgroups did. Meta-analyses based on multiple clinical trials also showed that combination therapy for ES-SCLC patients with brain metastases was not superior to chemotherapy alone (25, 26), consistent with our findings. Additionally, Jin et al.’s study indicated that pleural metastasis remains the predominant mode of failure following first-line chemotherapy plus ICIs for ES-SCLC, suggesting that thoracic radiotherapy might be an effective strategy (27).

Our study demonstrated no significant difference in outcomes between SCLC cases treated with PD-1 inhibitors and those treated with PDL-1 inhibitors when combined with chemotherapy and ICIs. Although the mOS and mPFS were slightly longer for PD-1 inhibitors, no direct clinical trials have compared these two types of drugs. A network meta-analysis that synthesized data from multiple randomized controlled trials found no significant differences in OS (HR 0.96, 95% CI: 0.72-1.30), PFS (HR 0.83, 95% CI: 0.72-1.30), or ORR (OR 1.39, 95% CI: 0.66-2.50) between PD-1 inhibitor + chemotherapy and PDL-1 inhibitor + chemotherapy. Bayesian ranking analysis suggested a trend toward longer OS with PDL-1 inhibitor + chemotherapy compared to PD-1 inhibitor + chemotherapy (28). Similarly, Pillai et al.’s study on NSCLC indicated that the efficacy and toxicity profiles of PD-1 and PDL-1 inhibitors appeared similar, although immune-related adverse events were slightly more frequent in patients receiving PD-1 inhibitors (29). The question of whether PD-1 and PDL-1 inhibitors result in different clinical outcomes in SCLC remains unresolved, and further studies are warranted.

Our multivariate analysis revealed that a poor ECOG PS (1 *vs* 0) and brain metastases were independent prognostic factors for PFS in ES-SCLC patients treated with chemotherapy plus ICIs. Previous studies have demonstrated that an ECOG PS of 3–4 versus 0–1 is independently associated with long-term prognosis in SCLC patients receiving first-line chemotherapy (30). However, despite Phase III clinical trials excluding patients with ECOG PS ≥2, one study indicated that chemotherapy plus ICIs could extend PFS in patients with ECOG PS 2 or 3 extensive-stage SCLC (31). Subgroup analysis within this study, however, showed no benefit for patients with ECOG PS 1 from chemotherapy plus ICIs, possibly due to inconsistencies in the control group. Moreover, when comparing the efficacy between ECOG PS 2–3 and ECOG PS 0–1 in SCLC patients undergoing chemotherapy plus ICIs, no significant differences were observed in PFS or OS (32). In patients with ES-SCLC and an ECOG PS less than 2, an ECOG PS of 1 compared to 0 was identified as an independent risk factor for PFS (33), which aligns with our findings, and a potential explanation is that the physical condition significantly influences the development of the tumor (34).

Brain metastases are among the most frequent sites of distant spread in ES-SCLC, and patients diagnosed with brain metastases typically face a less favorable prognosis. Whole-brain radiotherapy remains the standard treatment for ES-SCLC brain metastases. Consistent with several clinical trials, including the CASPIAN trial (HR=0.69, 95% CI: 0.35-1.31) (23) and the ETHER701 trial (HR=0.64, 95% CI: 0.29-1.41) (35), our study found that ES-SCLC patients with brain metastases did not benefit from immunotherapy. Furthermore, a real-world study revealed that patients treated with PD-1/PD-L1 inhibitors showed better control of intracranial disease and experienced a reduced rate of intracranial progression in comparison to ES-SCLC patients who received only chemotherapy (36). This may be attributed to the active immune microenvironment in SCLC brain metastases: a study of 32 SCLC brain metastasis samples revealed that 93.8% had lymphocyte infiltration, 75% expressed PDL-1, and 34.4% had PDL-1 expression exceeding 5% (37). Retrospective studies have also indicated that ES-SCLC patients with brain metastases who received immunotherapy in addition to chemotherapy and radiotherapy had significantly higher PFS compared to those treated with chemotherapy plus radiotherapy alone (38). In this study, baseline brain metastases in ES-SCLC patients receiving chemotherapy plus ICIs were identified as an independent prognostic factor for PFS, underscoring the importance of monitoring brain metastases in ES-SCLC patients prior to chemotherapy plus ICIs. Furthermore, Li et al.’s study showed that the combination therapy of albumin-bound paclitaxel effectively treated ES-SCLC patients with brain metastases, achieving an ORR of 36.6% (39). Additional research is warranted to explore more effective treatment options.

Inflammation greatly contributes to the development and progression of cancer, impacting every stage of tumorigenesis. Within this process, cancer cells interact complexly with neighboring stromal and inflammatory cells to form the inflammatory tumor microenvironment (TME) (40). Several novel inflammatory and nutritional biomarkers, including the NLR, LMR, SIRI, and SI, have emerged as significant prognostic indicators in various cancers such as cervical (41), esophageal (42), gastric (43), and breast cancer (44). These markers provide valuable insights into disease progression and patient outcomes. However, the prognostic significance of these indicators in patients with ES-SCLC undergoing immunotherapy remains unclear. Our univariate analysis revealed that baseline NLR > 4, SIRI > 2.63, and SII > 146.11 were significantly associated with LMR > 2.01 in patients with ES-SCLC treated with chemotherapy plus ICIs. Multivariate analysis further indicated that a high SIRI was a protective factor for OS.

This finding is inconsistent with the results of some studies. Yilmaz et al. demonstrated that in ES-SCLC, multivariate analysis identified high SIRI as a risk factor for both PFS and OS prognosis; however, this study did not differentiate between various treatment modalities (45). In LS-SCLC patients undergoing combined radiotherapy plus ICId, a high SIRI was identified as an independent risk factor for OS (46). Conversely, in ES-SCLC patients treated with chemotherapy plus ICIs, a high SIRI was not associated with OS prognosis (33). These inconsistencies suggest that SIRI’s role varies across different stages of SCLC and treatment methods, potentially due to the impact of immunotherapy on the TME. In solid tumors, monocytes predominantly develop into immunosuppressive tumor-associated macrophages (TAMs), which results in an anti-tumor immune reaction that strongly complements immune checkpoint inhibition (47). Therefore, in the context of immunotherapy, monocyte differentiation and immunotherapy may exhibit synergistic effects. However, further research is needed to confirm these findings.

There are several limitations in this study that warrant consideration. 1, one of the major limitations of this study is the relatively small sample size (n = 74). Despite making every possible effort to ensure the representativeness of the sample and the quality of the data during the design phase, the small sample size might impact the statistical power and external validity of the results. To evaluate whether our research design has sufficient statistical power, we performed a retrospective power calculation. Nevertheless, due to the absence of a formal power analysis during the design stage, we were unable to report specific power values. Under these circumstances, we suggest that future studies carry out detailed power calculations during the design phase to guarantee an adequate sample size for detecting the expected effect size.

2, the relatively short follow-up period may have influenced the results; therefore, longer-term follow-up studies are necessary to validate these findings.

Given that ICIs therapy has only recently been included in the diagnosis and treatment guidelines in China, future studies with larger sample sizes are required to draw more robust conclusions.

3, this study employed a retrospective design. Although PSM was used to balance baseline characteristics, some residual confounding bias may still exist.

## 5 Conclusion

In summary, our findings indicate that incorporating immunotherapy into first-line chemotherapy significantly improves PFS and OS in ES-SCLC patients, while maintaining safety. In addition, poor ECOG PS, brain metastases, and high SIRI at baseline may serve as valuable prognostic indicators for disease progression and survival in ES-SCLC patients. These factors provide a reliable reference for clinicians to assess ES-SCLC patients outcomes in the context of chemotherapy plus ICIs treatment regimens. It is worth noting that these findings should be interpreted as hypothesis-generating, not definitive clinical conclusions.

## Data Availability

The original contributions presented in the study are included in the article/**Supplementary Material**. Further inquiries can be directed to the corresponding authors.

## Supplementary material

Supplementary Figure 1 Comparison of data before and after propensity score match (PSM).

Supplementary Figure 2 Love plot before and after PSM.
