# Supplementary material for: Efficacy and safety of first-line chemotherapy combined with immune checkpoint inhibitors for extensive-stage small cell lung cancer patients: a real-world propensity score matching study
Source: Front Immunol. 2025 Aug 13;16:1562458. doi: 10.3389/fimmu.2025.1562458 (PMC12380824; doi:10.3389/fimmu.2025.1562458)
Supplement: Supplementary Figure 1 — Comparison of data before and after propensity score match (PSM). [file DataSheet1.docx]

Supplementary material


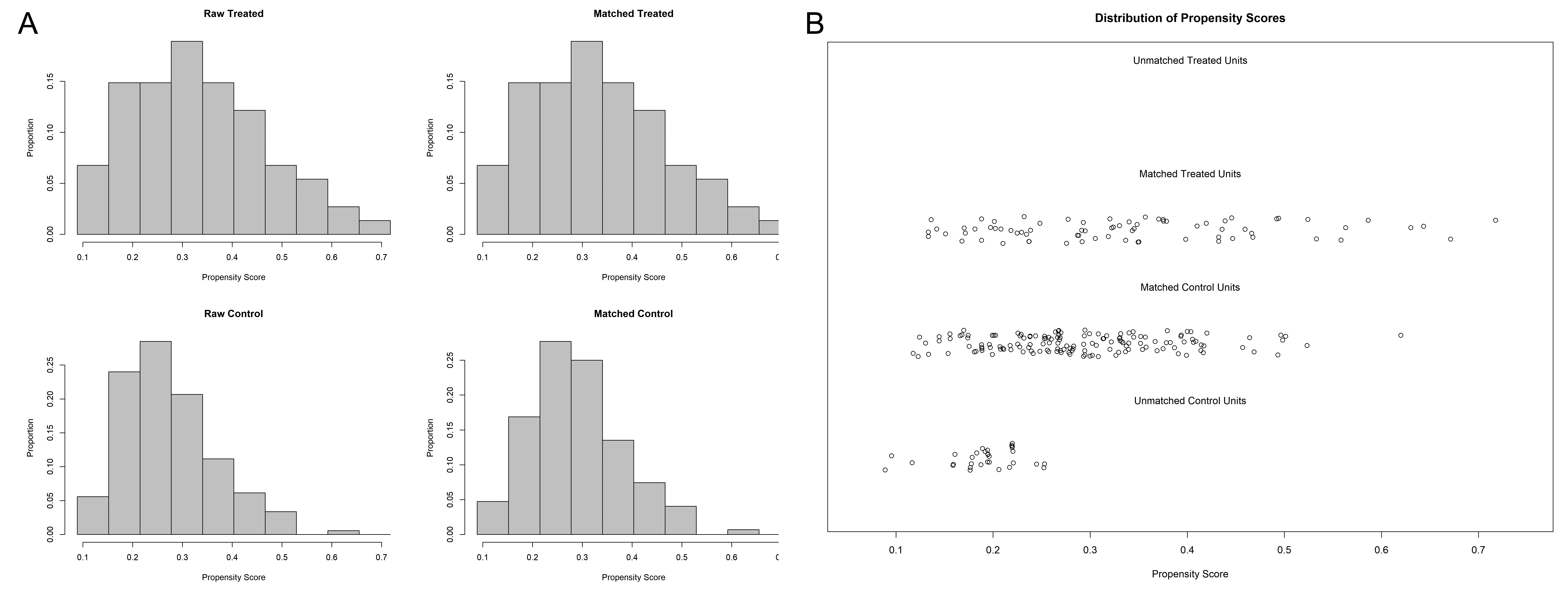


Figure S1 Comparison of data before and after propensity score match (PSM)


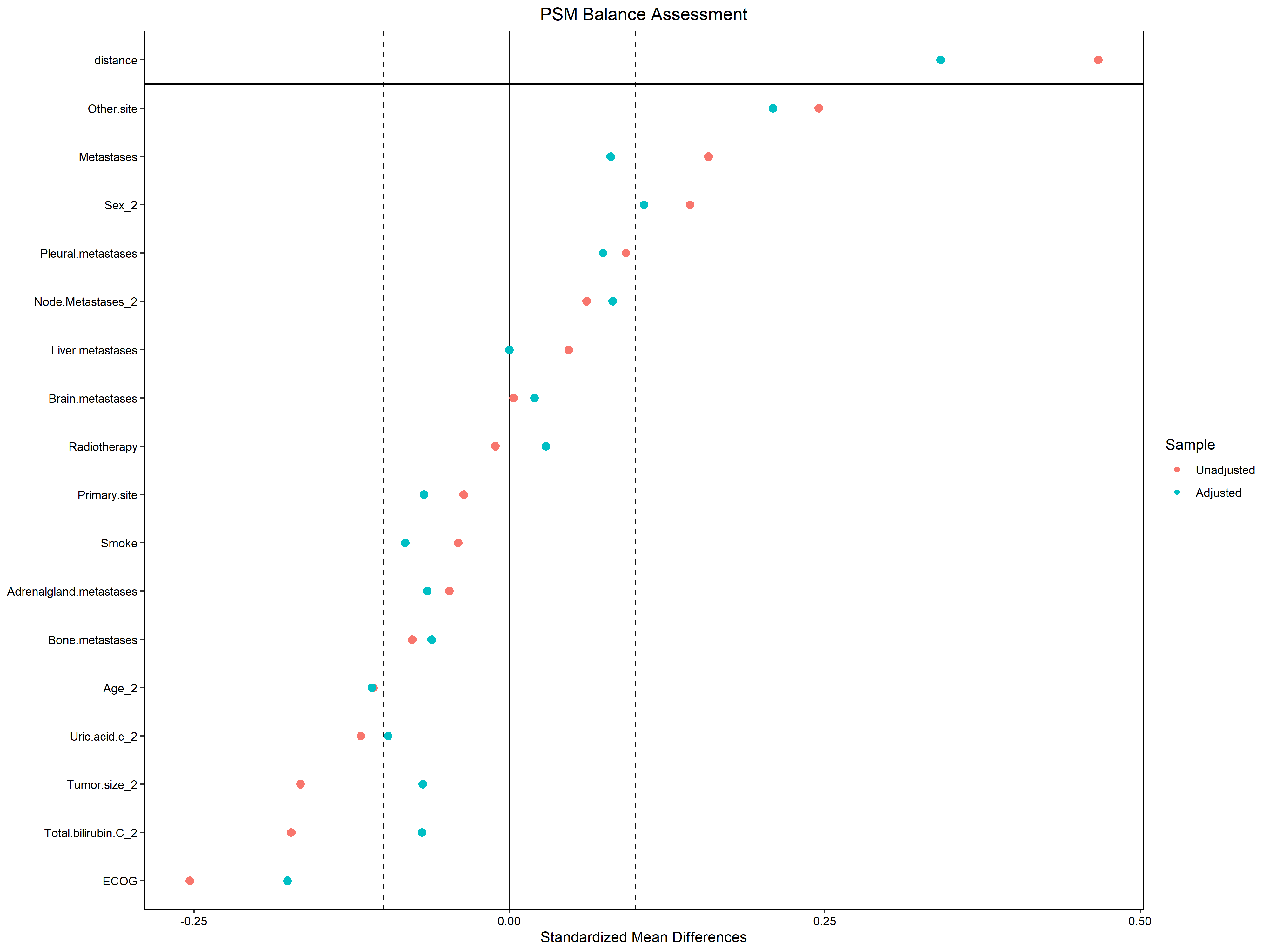


Figure S2 Love plot before and after PSM

Table S1 the SMD of different variables before and after PSM

| Variable | SMD -before PSM | SMD -after PSM |
| --- | --- | --- |
| Overall | 0.4669 | 0.3418 |
| Age | -0.0536 | -0.0541 |
| Sex | 0.0636 | 0.0473 |
| Smoke | -0.0199 | -0.0405 |
| ECOG PS | -0.1266 | -0.0878 |
| Primary site | -0.0181 | -0.0338 |
| Radiotherapy | -0.0053 | 0.0135 |
| Tumor size | -0.0814 | -0.0338 |
| Node metastases | 0.0302 | 0.0405 |
| Metastases | 0.0664 | 0.0338 |
| Adrenalgland metastases | -0.0148 | -0.0203 |
| Brain metastases | 0.0011 | 0.0068 |
| Liver metastases | 0.0212 | 0.0000 |
| Bone metastases | -0.0337 | -0.0270 |
| Pleural metastases | 0.0252 | 0.0203 |
| Other site | 0.0872 | 0.0743 |
| Total bilirubin | -0.0507 | -0.0203 |
| Uric acid | -0.0496 | -0.0405 |

Note: SMD, standardized differences; ECOG PS, Eastern Cooperative Oncology Group performance status.

Table S2 multiplicity adjusted (FDR) of subgroup

| Variable | | OS P value-adjusted | PFS P value-adjusted |
| --- | --- | --- | --- |
| Overall |  | <0.001 | <0.001 |
| Age | <65 | 0.100 | 0.052 |
|  | ≥65 | 0.006 | <0.001 |
| Sex | Male | 0.006 | <0.001 |
|  | Female | 0.100 | 0.055 |
| Smoke | NO | 0.011 | <0.001 |
|  | Yes | 0.046 | 0.040 |
| ECOG | 0 | 0.359 | <0.001 |
|  | 1 | <0.001 | 0.380 |
| Primary site | Left | 0.025 | 0.015 |
|  | Right | 0.025 | <0.001 |
| Radiotherapy | No | <0.001 | <0.001 |
|  | Yes | 0.174 | 0.027 |
| Tumor size | <5cm | 0.100 | 0.014 |
|  | ≥5cm | 0.006 | 0.014 |
| Node metastases | No | 0.100 | 0.041 |
|  | Yes | 0.006 | <0.001 |
| Metastases | No | 0.939 | 0.583 |
|  | Yes | <0.001 | <0.001 |
| Adrenalgland metastases | No | 0.006 | <0.001 |
|  | Yes | 0.086 | 0.077 |
| Brain metastases | No | 0.006 | <0.001 |
|  | Yes | 0.332 | 0.516 |
| Liver metastases | No | 0.006 | 0.009 |
|  | Yes | 0.163 | 0.014 |
| Bone metastases | No | 0.006 | 0.009 |
|  | Yes | 0.086 | 0.015 |
|  |  |  |  |
| Pleural metastases | No | <0.001 | <0.001 |
|  | Yes | 0.855 | 0.264 |
| Other site | No | <0.001 | <0.001 |
|  |  | 0.815 | 0.054 |

Note: FDR (false discovery rate), OS, Overall survival; PFS, progression free survival; ECOG PS, Eastern Cooperative Oncology Group performance status.
